# Effects of foliar application of humic acid extracts and indole acetic acid on important growth indices of canola (*Brassica napus* L.)

**DOI:** 10.1038/s41598-022-21997-5

**Published:** 2022-11-21

**Authors:** Arash Hemati, Hossein Ali Alikhani, Mehdi Babaei, Ladan Ajdanian, Behnam Asgari Lajayer, Eric D. van Hullebusch

**Affiliations:** 1 Department of Soil Science, Faculty of Agriculture, University of Tabriz, Tabriz, Iran; 2 Department of Soil Science, University College of Agriculture and Natural Resources, University of Tehran, Tehran, Iran; 3 Department of Horticultural Sciences, Faculty of Agriculture, Ferdowsi University of Mashhad, Mashhad, Iran; 4 Université Paris Cité, Institut de Physique du Globe de Paris, CNRS, 75005 Paris, France; 5 CEO of Qizil Topraq Sahand Company, Maragheh, Iran

**Keywords:** Plant sciences, Plant physiology

## Abstract

Vermicompost (VC) is a rich source of HA that improves plant growth and yield indices such as fresh and dry weights, plant height, stem diameter, leaf area, and chlorophyll index value. In this study, the effect of foliar application of HA extracted from different types of VC enriched with bacteria and/or fertilizers, commercial HA (CHA) and indole acetic acid (IAA) on the growth characteristics of canola (*Brassica napus*) in greenhouse conditions were compared. According to the results, the foliar application of HA extracted from VC had complete superiority over CHA and IAA in most traits except for the leaf number. Furthermore, the highest level of foliar application of HA (600 mg L^−1^) enriched with *Azotobacter chroococcum* (21Az) + *Pseudomonas fluorescens* (Ps 59) (HA-AS) generated the highest height, diameter, leaf area, and chlorophyll index value. Also, the highest stomatal conductance and photosynthesis rate were observed with the application of 600 mg L^−1^ HA extracted from VC enriched with nitrogen, sulfur, and phosphorus (HA-NSP) compared to the other treatments. Besides, dry and fresh weights and seed yield under HA-NSP and HA-AS treatments were at their highest rate. Among the extracted HAs, the one extracted from the nitrogen enriched VC had the lowest efficiency. Based on the present study, the HA extracted from VC enriched with *Azotobacter*, *Pseudomonas* and NSP is recommended to increase canola growth and production.

## Introduction

After some cereals [e.g. wheat (*Triticum aestivum*), rice (*Oryza sativa*), maize (*Zea mays*), oat (*Avena sativa*) and barley (*Hordeum vulgare*)], the second most important food source in the world are oilseeds^1^. Among these oilseeds (rich in fatty acids and protein), canola (*Brassica napus*) is one of the most important ones existing in the human food basket and the third largest source for producing vegetable oil in the world^2,3^. The use of natural and organic inputs in agriculture, including biofertilizers, plays an important role in increasing crop yield^4,5^. Although chemical fertilizers are one of the main factors in increasing soil fertility^6,7^, excessive use especially when combined with poor agricultural practices such as burning crop residues, drastically reduces the amount of soil organic matter^8^. The reduction of organic matter has a special effect on biological, chemical, and physical soil properties and increases the possibility of erosion^9^. Today, improper and prodigal use of chemical fertilizers, which has an adverse effect on soil structure, leads to imbalances in physical and chemical soil properties, resulting in reduced nutrient uptake and reduced crop yield^10,11^. As a result, due to the above-mentioned problems and also because of the increasing importance of environmental issues, more attention has been paid to biological fertilizers as replacement of chemical fertilizers^12^. Vermicompost (VC) is a plant growth stimulant organic fertilizer that, as a rich source of humic acid (HA), can improve the growth and yield indices of a wide range of plants^13^. HA extracted from VC, on the other hand, is often competitive with commercial forms, usually derived from coal or leonardite, and even with indole phytohormones (particularly, indole acetic acid—IAA)^14,15^. Many studies have been done on HA and fulvic acid, and positive correlation between plant growth and HA application have been seen^16,17^, but more and more detailed studies are still needed. HA is biochemically the active ingredient in humus, and it increases the biomass of roots and aerial organs through hormonal effects and improved nutrient uptake^18^. The aerial part of the plant provides phloem sap for root growth and the role of the root is to provide raw materials (e.g. water, mineral nutrients and hormones); therefore, there is a reciprocal relationship between the root and the aerial part^19^. In fact, root growth is affected by the environment and factors such as moisture, temperature and soil nutrients^20^. HA and fulvic acid form stable and insoluble complexes and soluble complexes with microelements, respectively, and increase nutrients uptake, soil fertility, and production in plants^21^. One of the most important components of soil can be HA that can easily improve the availability of nutrients in soils and affect other important chemicals, biological and physical soils parameters. Foliar application of HA at concentrations of 0, 0.1, and 0.2% was reported to increase the uptake of nutrients in the corn (*Zea mays*) plant. Also, the highest concentration of foliar application generated the highest dry weight and yield^22^. The effects of foliar application of humic substances extracted from leonardite on the growth of olive (*Olea europaea*) cuttings were investigated by Fernandez-Escobar et al.^23^. Application of concentrations used in the study in doses of 0.5% and 1% improved the growth of olive cuttings. Also, foliar application of leonardite extracts had a significant effect on the nutrient concentration of olive leaf cuttings^23^. The effect of HA foliar application on photosynthesis or stomatal conductance in the wheat plant was not observed, but it had an effect on Rubisco activity and leaf protein content, and nitrogen distribution in soil^24^. Foliar application of HA for a long time (8 weeks) has been reported to have a positive effect on fruit quality, reduce the number of deformed and rotten fruits and increase the sugar content in strawberries (*Fragaria x ananassa* L.). According to Neri et al.^25^ reports, these positive effects on fruit quality are probably due to the positive physiological effect of HA foliar application on the whole plant and increase plant efficiency. As a result, in view of the importance of canola in oil extraction and also the approach of the international community dedicated to the protection of natural resources in order to reduce the use of chemical fertilizers, the use of biofertilizers, including HA, in the present study, was established and implemented.

In this regard, the foliar application of HA extracted from VC on canola was investigated. In general, the use of HA, in addition of not generating advert effect on the environment, can play a positive role in increasing the yield of most plants and can be used as a substance of natural origin to reduce the use of chemical fertilizers and increase crop production. On the other hand, the quasi-hormonal properties of some compounds encountered in HA are expected to stimulate plant growth, so their application in different concentrations and enrichment with different substances can have different effects that have not been tested before in such an approach and therefore contribute to the novelty of this research.

## Materials and methods

### VC production

VC was produced from cow manure raw materials and plant residues in a 1:3 ratio (weight:weight) in the presence of composting worms (*Eisenia fetida*) for a period of 5 months at the VC Education and Research Station of Agriculture and Natural Resources Campus in Tehran University. To this aim, first cow manure and plant residues were placed under sunshine for 1 month; then, small dome-shaped hills with a width of 70, length of 200, and height of 50 cm were formed. Following sufficient irrigation monitored till the occurrence of leachate, *Eisenia fetida* worms were inserted into the bed (500 earthworms per 100 kg of bed). The humidity of the hills was approximately 50–60% during the composting period, and the humidity was maintained through daily irrigation. By the end of the processing period, earthworms were separated from the final product (VC). The characteristics of the VC were analyzed according to Gupta^26^ and the data are presented in Table 1.

**Table 1:** Initial vermicompost properties.

pH	EC (dS m^−1^)	Total N (%)	OC (%)	P (%)	K (%)	Na (%)	Fe (%)	Ca (%)	C/N
7.63	2.14	1.2	24.37	0.82	6.52	1.1	0.57	8.5	20.3

### VC enrichment treatments

In order to enrich VC with bacterial treatments, phosphate solubilizing bacteria (*Pseudomonas*) and nitrogen-fixating bacteria (*Azotobacter*) were added. *Azotobacter* and *Pseudomonas* belong to the *Azotobacter chroococcum* and *Pseudomonas fluorescens s*pecies, respectively. Both strains were obtained from the beneficial terricolous microorganism gene bank of the department of soil science engineering at Tehran University where the strains have been isolated, identified, and maintained^27^. After the second bacterial enrichment, the inoculated bacterial population was adjusted at 4 × 10^9^ cfu (Colony Forming Unit) mL^−1^ and 25 mL of each liquid enrichment was used for inoculation per 1 kg of VC^28^. In addition, it was attempted for this study to make use of necessary chemical elements (nitrogen, phosphorus, and sulfur) as fertilizer treatments to enrich VC. One percent of each element was added to VC, the source of added nitrogen being urea, source of phosphorus being triple superphosphate and source of sulfur being elemental sulfur. Potassium was not added due to the richness of potassium in the produced VC. Prior to enrichment, VC samples were screened using a 2 mm sieve and their large particles were separated.

### Extraction of HA

Humic acid was extracted from vermicompost according to the procedure described by Qi et al.^29^. For this purpose, VC samples (the VC samples were provided by the VC production station of the Agriculture and Natural Resources Campus of University of Tehran) were mixed with a ratio of 1:10 (liquid/solid) with 0.5 M of sodium hydroxide and were shaken for a period of one, seven and nine days (extraction time) at 160 rpm in a dark room. The soluble phase was separated from the solid fraction by centrifugation (4000*g*) and its pH was reduced to less than 2 with the use of 6 M of HCl to precipitate HA and separate it from fulvic acid. The separated HA was purified with HCl/HF (0.1 M of HCl and 0.3 M of HF), washed with distilled water until the pH reached about 4–5, and finally dried at a temperature below 50 °C. Some chemical properties of CHA and extracted HA were measured and are presented in Table 2. Briefly, Total acidity and carboxyl groups were measured according to Page's proposed method^30^, and also OH-phenolic functional groups were calculated from the difference between total acidity and carboxyl groups. HA percentage was measured in experimental treatments by Qi et al.^29^. According to Table 2, CHA had less functional groups than HA extracted from VC.

**Table 2:** The average comparison table related to the results of the effect of different enrichment treatments on the values of functional groups in HA.

Treatments	Total acidity (mmol g^−1^)	Carboxylic groups (mmol g^−1^)	OH-phenolic groups (mmol g^−1^)	Percentage of pure HA
CHA	4.5	2.8	1.7	75
VCA	5.67	3.41	2.25	85
VC-AS	6.82	4.14	2.68	85
VC-N	6.32	3.22	3.09	85
VC-NSP	6.81	4.10	2.71	85

### Plant materials and foliar spray application level

Six enriched treatments were sprayed on plant, 1—HA extracted from VC without enrichment (HA-A); 2—HA extracted from VC enriched with 1% nitrogen (HA-B); 3—HA extracted from VC enriched with 1% nitrogen, 1% sulfur, and 1% phosphorus (HA-D); 4—HA extracted from VC enriched with *Azotobacter chroococcum* (21Az) + *Pseudomonas fluorescens* (Ps 59) (HA-AS); 5—Commercial humic acid (CHA) was prepared from 75% pure CHA from leonardite imported from China (Hebei China Company) was obtained from the market and 6—Indole-3-acetic acid (IAA) was provided by the University of Tehran (Forbes Pharmaceuticals Company). Additionally, the CHA and HA at 4 concentration levels of 0, 200, 400, and 600 mg L^−1^ and IAA acid with concentrations of 10^–6^, 10^–5^, and 10^–4^ molar (Table 3) were applied in five periods at 10, 20, and 30-day stages after seedling as well as at flowering and reproductive stages. Totally, each treatment was dissolved in distilled water at 10–30 mL depending on the application time after seedling stages. So that, volumes of 10, 20 and 30 mL were used to 10–30 days seedling, flowering and reproductive stages, respectively. Then it was sprayed on each pot. Each pot contained two plants. Different volumes of suspensions used at different times of foliar spraying and different treatments were used, and the criterion for its application was wetting the whole plant in such a way that water droplets dripped from the aerial parts.

**Table 3:** Application level [1—HA extracted from VC without enrichment (HA); 2—HA extracted from VC enriched with 1% nitrogen (HA-N); 3—HA extracted from VC enriched with 1% nitrogen, 1% sulfur, and 1% phosphorus (HA-NSP); 4—HA extracted from VC enriched with *Azotobacter chroococcum* (21Az) + *Pseudomonas fluorescens* (Ps 59) (HA-AS); 5—Commercial humic acid (CHA); 6—Indole-3-acetic acid (IAA)].

Level 1	Level 2	Level 3	Level 4
No additive of HA	200 mg L^−1^ HA	400 mg L^−1^ HA	600 mg L^−1^ HA
No additive of HA-N	200 mg L^−1^ HA-N	400 mg L^−1^ HA-N	600 mg L^−1^ HA-N
No additive of HA-NSP	200 mg L^−1^ HA-NSP	400 mg L^−1^ HA-NSP	600 mg L^−1^ HA-NSP
No additive of HA-AS	200 mg L^−1^ HA-AS	400 mg L^−1^ HA-AS	600 mg L^−1^ HA-AS
No additive of IAA	10^–6^ molar of IAA	10^–5^ molar of IAA	10^–4^ molar of IAA
No additive of CHA	200 mg L^−1^ CHA	400 mg L^−1^ CHA	600 mg L^−1^ CHA

### Pot experiments

Pots were placed in the greenhouse environment after being prepared. The growing stage was carried out by manually placing five shrubs in plastic pots containing 3 kg of soil. The Loamy soil used in this study was collected from the research farm of Karaj Soil and Water Institute located in Meshkin Dasht, Karaj (Iran). Sampling was performed from a depth of 0–30 cm of the soil surface and the samples were transferred to the laboratory. Pots were filled with air-dried soil samples that were passed through a 4 mm sieve. Some soil was passed through a 2 mm sieve for laboratory operations. Some physicochemical properties of the soil sample were measured after air drying, crushing, and passing through a 2-mm sieve and presented in Table 4. Overall, available-P by Olsen method, total nitrogen by Kjeldahl method, available-K by 1 N acetate ammonium, available Zn and Fe by DTPA-TEA, EC and pH in saturated extract, soil texture by hydrometric method were measured^30,31^. Seeds of canola were obtained from Oilseed and Plant Improvement Institute of Karaj, Iran. The Oilseed and Plant Improvement Institute declared that seeds of canola were obtained under national and international guidelines and the seed were prepared under the supervision and permission of University of Tehran and all authors comply with all the local and national guidelines. The voucher specimens of the plants were deposited at the herbarium of Department of Horticultural Sciences, University of Tehran, Karaj, Iran. After germination and complete settlement of plant germs, their numbers were narrowed down to two per pot. In this study, the modified RGS (Spring cultivar and sensitive to cold) canola cultivar was used which was obtained from Karaj Seed and Seedling Research Institute. The minimum and maximum temperatures of the greenhouse was between 20 and 28 °C, respectively, with a relative humidity of 75–80%. In addition, the canola seedlings were exposed to 14 h of light (a combination of fluorescent and tungsten lamps), daily.

**Table 4:** Physical and chemical properties of experimental soil.

Electrical conductivity, dS m^−1^	pH	Zn, mg kg^−1^	Fe, mg kg^−1^	P, mg kg^−1^	K, mg kg^−1^	Nitrogen, mg kg^−1^	Clay, %	Sand, %	Silt, %
1.6	7.2	0.74	2.5	8.1	370	800	33	39	28

### Plant growing stage

The plants growth period was completed within 4 months during which the pots were visited daily, and the humidity of each pot were adjusted at 0.75–0.8 field capacity in terms of weight. The harvesting stage began by the end of the growth period, after the plants grew clusters.

### Harvesting and grinding stage

After harvesting and weighing the wet weight of aerial organs, these organs were washed entirely using distilled water; next, they were placed inside clean paper envelopes and then dried in an oven for 48 h at 65 °C; subsequently, the dry weight of the aerial organ was measured as well. Then, the dried aerial organ was separately powdered using a grinder and then placed inside lidded containers to produce herbal extracts and perform analytical experiments. Furthermore, the root system of the plant was completely taken out of the soil as much as possible and then placed in a basin full of water; next, the surrounding soil was washed away and ultimately, the wet and dry weights of the root were determined^3^.

### Measuring plant characteristics after harvesting

The height of the shrub was measured from crown to the tip of the stamen in centimeters, without taking the root into account. The root was carefully separated from the soil and washed with water to remove its adhered soil as much as possible. After separating the root from the soil, its length was measured in centimeters using a ruler. All leaves for each shrub were counted during the growing and harvesting periods. The length of the largest inflorescence in each shrub was measured in centimeters from the stem growth location. The length of the largest inflorescence was solely measured due to difference in the number of inflorescences under various treatments; moreover, measuring the lengths of all inflorescences and indicating inflorescence average length would not have represented the reality of the study in a few cases. Stem diameter was measured in millimeters using calipers from the stem, under the first internode. To determine the extent of chlorophyll in a leaf, SPAD-502 manual chlorophyll meter (Minolta, Japan) was used without damaging plant textures and extracting from leaves. To this end, three leaves were selected on average during the flowering stage and the extent of chlorophyll was estimated from its central point. Leaf area was measured using a leaf area meter tool, model Delta T-Devices UK (ΔT Area Meter MK2). The extent of photosynthesis was measured in µM CO_2_ × m^−2^ × s^−1^. Stomatal conductance was measured via an aerial infrared gas analyzer (IRGA) tool, model LCA4-ADC. At the end of the 4-month period and after the clusters were formed and dried, the number of clusters, percentage of fertile clusters and the total seed weight were measured for each plant according to Eq. (1)^3^.

1 $${\text{Fertile Clusters }}\left( \% \right) \, = \, \left( {{\text{Number of Fertile Clusters}}/{\text{Total Number of Clusters}}} \right) \times {1}00$$

### Evaluating the amount of oil contents in canola

The oil existing inside the seeds was extracted using Soxhlet method, via methanol-chloroform organic solvent in a 1:2 ratios and three repetitions. The method was used on canola for the first time by Joshi et al.^32^. The dried seeds were powdered, transferred to M_3_ Whatman Filter Paper, and weighed (Weight A). The sample was then packed tightly and placed inside an oven for 6–8 h and was dried until reaching a stable weight (Weight B). After cooling at room temperature in a desiccator, samples were transferred to Soxhlet pipes and were extracted for 24 h via petroleum ether (b.p. below 50 °C). Following extraction, the packed samples were placed under a hood to evaporate the remaining petroleum ether and become dried; ultimately, desiccator was cooled and then weighed (Weight C). Oil contents (%) was calculated according to Eq. (2) ^3^.

2 $${\text{Oil}}\;{\text{content }}\left( \% \right) \, = \, \left( {{\text{B }}{-}{\text{ C}}} \right)/\left( {{\text{B }}{-}{\text{ A}}} \right) \times { 1}00\%$$

The dried seeds were triturated and transferred to 3 M Whatman filter paper and weighed (weight A), the sample was tightly closed and then dried in an oven for 6–8 h until a stable weight was reached (weight B). After cooling to room temperature in a desiccator, the samples were transferred to Soxhlet tubes and extracted with petroleum ether for 24 h (b.p. below 50 °C) then, after extracting the packaged samples for evaporation, the remaining petroleum ether was placed in the hood to dry and finally, the desiccator was cooled and then weighed (weight C). Three replicates were prepared for each sample and the average value of these three treatments was employed to calculate the amount of oil contents.

### Producing herbal extraction

The dry ashing method was employed to produce herbal extract. To this end, 1 g of ground dry plant material was poured into a crucible and then placed inside a furnace; temperature was gradually raised to 450 °C so that white ash is produced. After the samples were cooled, 20 mL of 2 N hydrochloric acids was added to each sample and then placed in a sand bath for 30 min. Finally, samples were filtered in 100 mL volumetric flask and brought to volume^33^.

### Total phosphorus analysis

In order to measure phosphorus, the yellow method (Molybdovanadate) was employed. Accordingly, the plant sample solution was prepared following the preparation of phosphorus yellow and standard solutions. First, 20 mL of the herbal extract produced using the dry ashing method was poured into a 100 mL volumetric flask; then, 20 mL of yellow indicator and 20 mL of distilled water were added. After 45 min, the solution was brought to volume and phosphorus contents was read using a spectrophotometer at 430 nm wavelength. Prior to reading plant samples, the standard solutions were read using the device and its chart was obtained^33^.

### Measuring copper concentration in plant’s aerial organ

The concentration of copper in herbal extracts was measured and reported using an atomic absorption spectroscopy (Shimadzu AA-670). For this assay, 0.1 g of the dried plant organs of each pot were digested with 2 mL of 60% nitric acid overnight and then placed in a water bath for 2 h at 90 °C. After cooling, 1 mL of hydrogen peroxide was added to the samples and the tubes were placed in a water bath at 90 °C for half an hour. After cooling, the samples were reduced to 10 mL with distilled water. Root copper and aerial parts of the plant were measured using a device during Shimadzu AA-670 atomic absorption spectrometry and a calibration curve was drawn with respect to the exclusive wavelength of each element; finally, samples were read^33^.

### Experiment design and statistical analysis

Greenhouse experiments were performed as a factorial in the form of randomized complete block design (RCBD) (with 2 factors including treatment and treatment level) in four repetitions. The obtained results were analyzed using SAS software and the related variance analysis tables were drawn. Additionally, comparison of data mean values was performed by using Duncan’s multiple range test at 5% level via MSTAT-C software^3^.

## Results

### Effects of amendment formulation on canola growth features

#### Morphological traits

**Figure 1:**
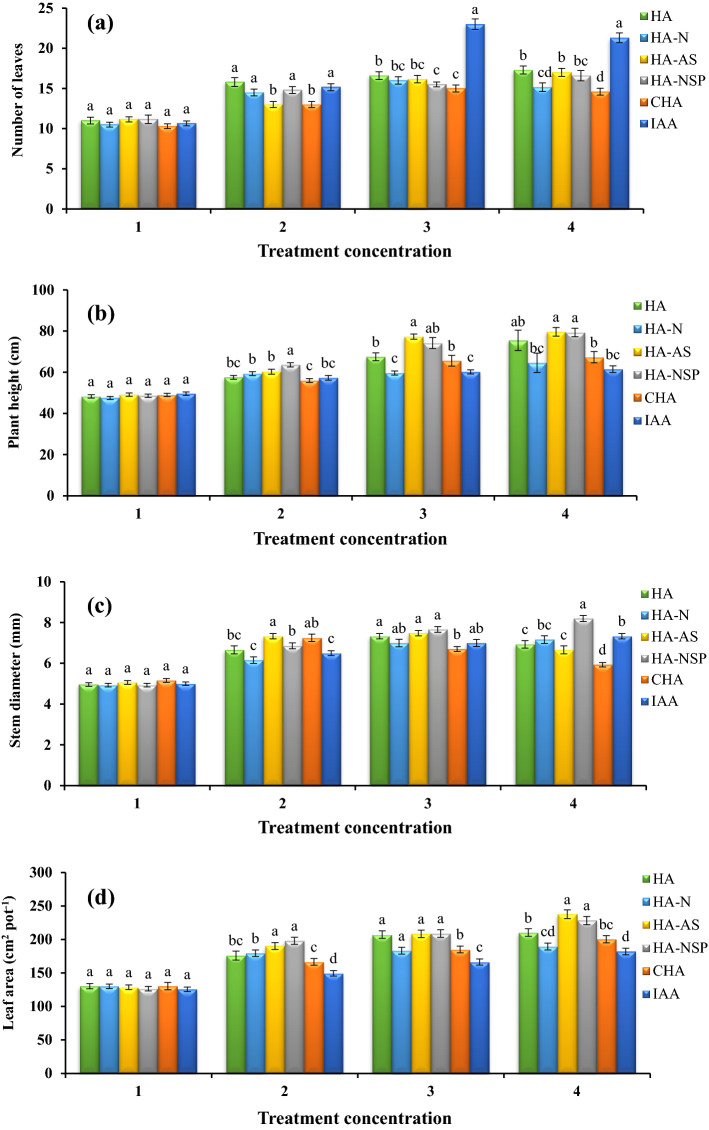
Comparison of average foliar application of experimental treatments on number of leaves (**a**), plant height (**b**), stem diameter (**c**) and leaf area (**d**) at treatment concentrations (levels 1, 2, 3 and 4 for HA, HA-N, HA-AS, HA-NSP and CHA treatments of 0, 200, 400 and 600 mg L^−1^ and 0, 10^–6^, 10^–5^ and 10^–4^ molar IAA). Different letters in each figure show significant difference at *p* ≤ 0.05 by Duncan multiple range test. HA, HA extracted from vermicompost without enrichment; HA-N, HA extracted from vermicompost enriched with 1% nitrogen; HA-NSP, HA extracted from vermicompost enriched with 1% nitrogen, 1% sulfur, and 1% phosphorus; HA-AS, HA extracted from vermicompost enriched with *Azotobacter chroococcum* (21Az) + *Pseudomonas fluorescens* (Ps 59); CHA, Commercial humic acid; IAA, Indole-3-acetic acid (IAA).

The effect of different amendment formulations used for foliar application on the morphological traits was significant at 1% probability level. The highest number of leaves (23) in canola was observed with the foliar application of IAA at the level of 10^–5^ molar, which had a significant advantage over other treatments (Fig. 1a). HA extracted from NPS-enriched VCs (HA-NSP) as well as HA extracted from *Pseudomonas* and *Aztobacter* enriched VC (HA-AS) generated the highest plant height (79 cm) at 600 mg L^−1^ (Fig. 1b), and HA extracted from NPS-enriched VC (HA-NSP) generated the highest canola stem diameter (8.2 mm) at the highest level, i.e., 600 mg L^−1^ (Fig. 1c) between all tested enriched treatments. Regarding the stem height and diameter, the foliar application of CHA as well as IAA was not efficient enough.

#### Leaf area and SPAD index

**Figure 2:**
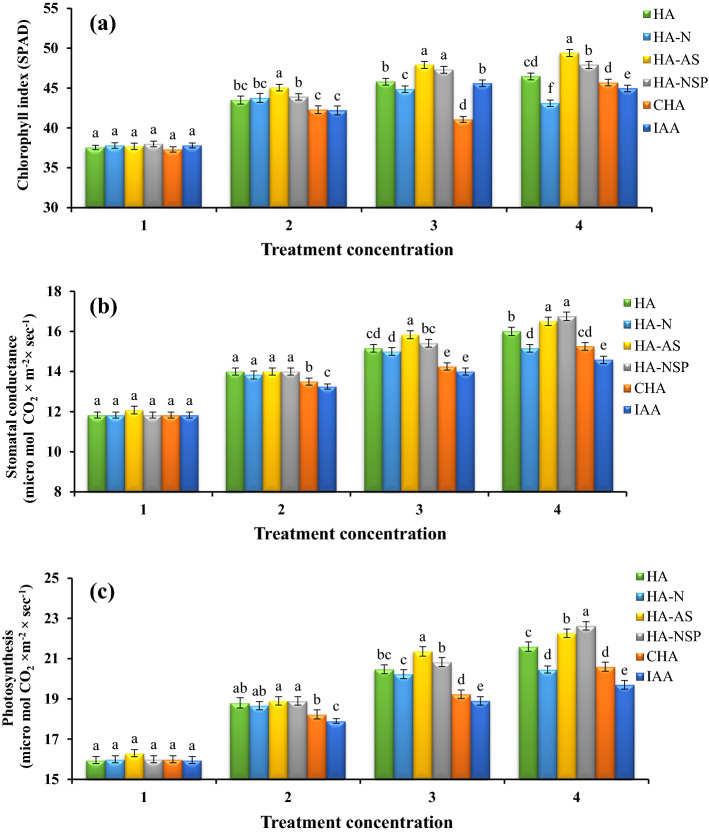
Comparison of average foliar application of experimental treatments on chlorophyll index (SPAD) (**a**), stomatal conductance (**b**), and photosynthesis (**c**) at treatment concentrations (levels 1, 2, 3 and 4 for HA, HA-N, HA-AS, HA-NSP and CHA treatments of 0, 200, 400 and 600 mg L^−1^ and 0, 10^–6^, 10^–5^ and 10^–4^ molar IAA). Different letters in each figure show significant difference at *p* ≤ 0.05 by Duncan multiple range test. HA, HA extracted from vermicompost without enrichment; HA-N, HA extracted from vermicompost enriched with 1% nitrogen; HA-NSP, HA extracted from vermicompost enriched with 1% nitrogen, 1% sulfur, and 1% phosphorus; HA-AS, HA extracted from vermicompost enriched with *Azotobacter chroococcum* (21Az) + *Pseudomonas fluorescens* (Ps 59); CHA, Commercial humic acid; IAA, Indole-3-acetic acid (IAA).

The results of the variance analysis of leaf area and SPAD index showed that there is a statistically significant difference between different levels of treatments for both traits. The results of comparing the means with the help of Duncan's new multiple range test at 5% probability level indicated that the highest values of these traits were obtained from the HA treatment extracted from the VC treatments. Among the enriched HA treatments, AS and D had higher values of these indices and A and B had lower values than the previous two treatments. Based on the results of means comparison, compared to the other treatments, canola leaf area and SPAD index, both at the level of 600 mg L^−1^, enriched with *Pseudomonas* and *Aztobacter* (HA-AS), were at the highest values of 237.67 cm^2^ per pot and 49.4 SPAD, respectively (Figs. 1d and 2a). Additionally, the use of CHA and IAA treatments as foliar application were not efficient enough in improving leaf area and SPAD value.

#### Stomatal conductance and photosynthesis

The results of mean comparison showed a significant difference between different treatments. The treatments of HA extracted from VC generated the highest rates for stomatal conductance and photosynthesis. Among the treatments of HA extracted from VC at 600 mg L^−1^, the highest amount of photosynthesis and stomatal conductance in HA enriched with NPS (HA-D) were observed to be 22.63 micro mol CO_2_ × m^−2^ × sec^−1^ and 16.75 micro mol CO_2_ × m^−2^ × sec^−1^, respectively (Fig. 2b,c).

### Effect of using different treatments on dry and wet weights of aerial organ and root

**Figure 3:**
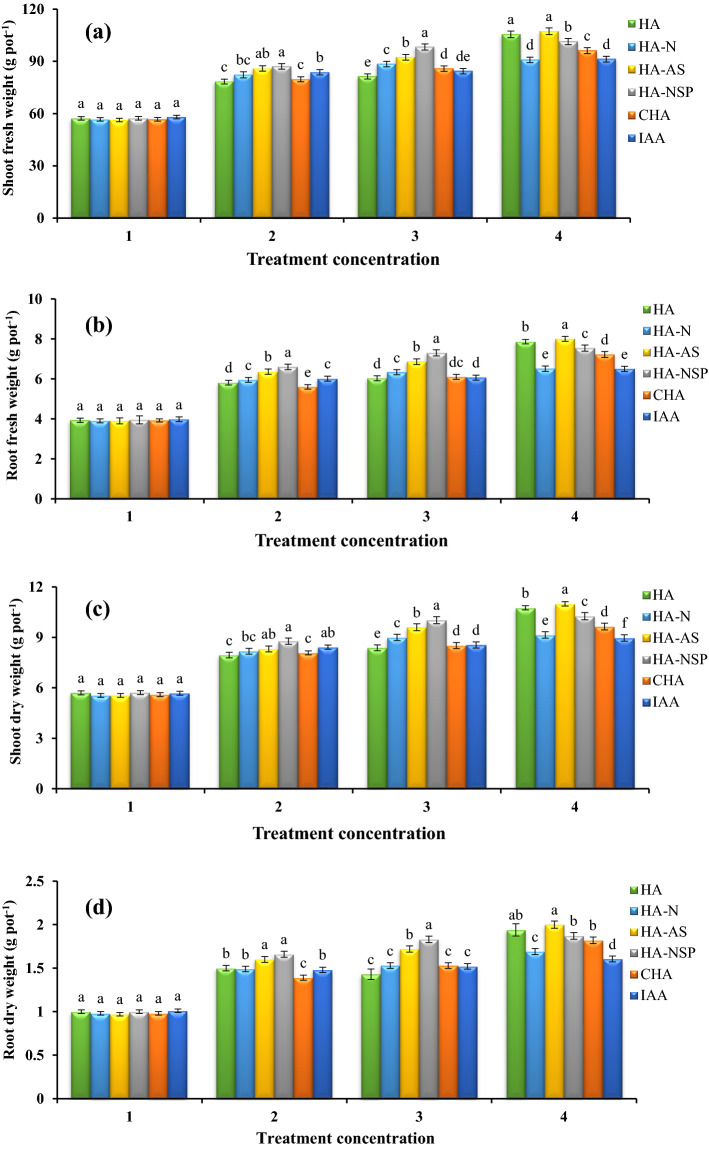
Comparison of average foliar application of experimental treatments on shoot fresh weight (**a**), root fresh weight (**b**), shoot dry weight (**c**) and root dry weight (**d**) at treatment concentrations (levels 1, 2, 3 and 4 for HA, HA-N, HA-AS, HA-NSP and CHA treatments of 0, 200, 400 and 600 mg L^−1^ and 0, 10^–6^, 10^–5^ and 10^–4^ molar IAA). Different letters in each figure show significant difference at *p* ≤ 0.05 by Duncan multiple range test. HA, HA extracted from vermicompost without enrichment; HA-N, HA extracted from vermicompost enriched with 1% nitrogen; HA-NSP, HA extracted from vermicompost enriched with 1% nitrogen, 1% sulfur, and 1% phosphorus; HA-AS, HA extracted from vermicompost enriched with *Azotobacter chroococcum* (21Az) + *Pseudomonas fluorescens* (Ps 59); CHA, Commercial humic acid; IAA, Indole-3-acetic acid (IAA).

The effect of the different treatment levels used for all the above-mentioned traits was significant at 1% probability level. The results of means comparison showed that the treatments of HA extracted from VC enriched with nitrogen, sulfur and phosphorus (HA-D) had the highest fresh and dry weight of aerial organs and roots at 600 mg L^−1^ level. The highest fresh weight of canola’s aerial organs was 107.3 g and the highest fresh weight of its roots was 8 g (Fig. 3a,b). Also, dry weight of aerial organs and roots were reported to be 11 and 2 g, respectively (Fig. 3c,d).

### Effect of using different treatments on seed performance, inflorescence lengths, and oil percentage in seeds

**Figure 4:**
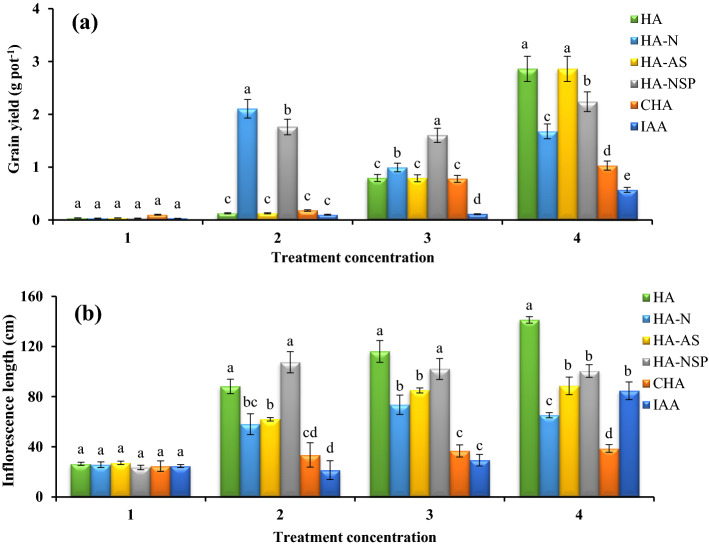
Comparison of average foliar application of experimental treatments on grain yield (**a**) and inflorescence length (**b**) at treatment concentrations (levels 1, 2, 3 and 4 for HA, HA-N, HA-AS, HA-NSP and CHA treatments of 0, 200, 400 and 600 mg L^−1^ and 0, 10^–6^, 10^–5^ and 10^–4^ molar IAA). Different letters in each figure show significant difference at *p* ≤ 0.05 by Duncan multiple range test. HA, HA extracted from vermicompost without enrichment; HA-N, HA extracted from vermicompost enriched with 1% nitrogen; HA-NSP, HA extracted from vermicompost enriched with 1% nitrogen, 1% sulfur, and 1% phosphorus; HA-AS, HA extracted from vermicompost enriched with *Azotobacter chroococcum* (21Az) + *Pseudomonas fluorescens* (Ps 59); CHA, Commercial humic acid; IAA, Indole-3-acetic acid (IAA).

**Figure 5:**
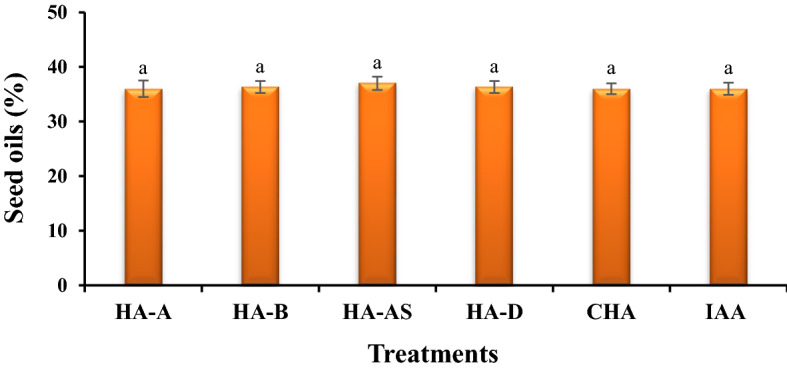
Comparison of average foliar application of experimental treatments on seed oils at different treatments. Different letters show significant difference at *p* ≤ 0.05 by Duncan multiple range test. HA, HA extracted from vermicompost without enrichment; HA-N, HA extracted from vermicompost enriched with 1% nitrogen; HA-NSP, HA extracted from vermicompost enriched with 1% nitrogen, 1% sulfur, and 1% phosphorus; HA-AS, HA extracted from vermicompost enriched with *Azotobacter chroococcum* (21Az) + *Pseudomonas fluorescens* (Ps 59); CHA, Commercial humic acid; IAA, Indole-3-acetic acid (IAA).

The comparison of mean results showed that the effect of treatments used was significant at 1% probability level, and the treatment of HA-AS at the level of 600 mg L^−1^ generated the highest Grain yield (2.86 g per pot) per plant between all treatments (Fig. 4a). Although IAA treatment had a large number of clusters, most of these clusters were empty and therefore had the lowest total seed weight per plant. The results of inflorescence length measurement showed that the highest length, which was 141.3 cm, was observed in the treatment of unenriched HA (HA-A) with the highest level of foliar application (Fig. 4b). In the analysis of oil percentage, no significant effect was observed between the measured treatments (Fig. 5).

### Effect of using different treatments on concentrations of phosphorus and copper in the aerial part of canola

**Figure 6:**
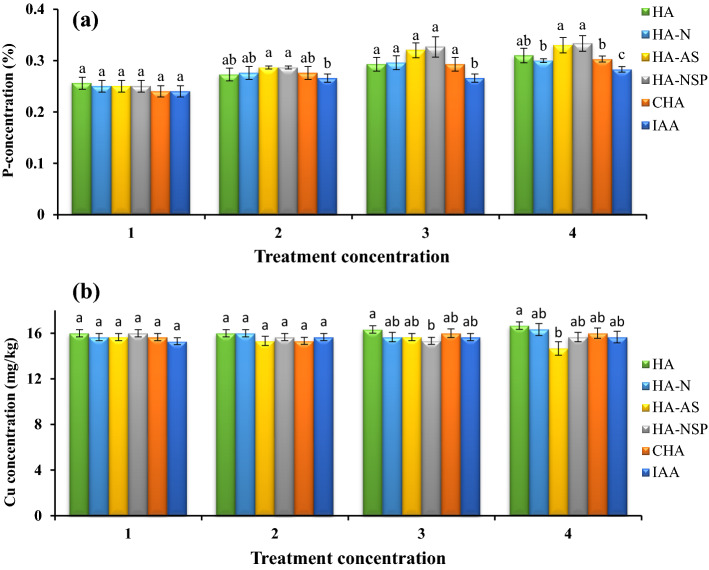
Comparison of average foliar application of experimental treatments on P (**a**) and Cu-concentrations (**b**) at treatment concentrations (levels 1, 2, 3 and 4 for HA, HA-N, HA-AS, HA-NSP and CHA treatments of 0, 200, 400 and 600 mg L^−1^ and 0, 10^–6^, 10^–5^ and 10^–4^ molar IAA). Different letters in each figure show significant difference at *p* ≤ 0.05 by Duncan multiple range test. HA, HA extracted from vermicompost without enrichment; HA-N, HA extracted from vermicompost enriched with 1% nitrogen; HA-NSP, HA extracted from vermicompost enriched with 1% nitrogen, 1% sulfur, and 1% phosphorus; HA-AS, HA extracted from vermicompost enriched with *Azotobacter chroococcum* (21Az) + *Pseudomonas fluorescens* (Ps 59); CHA, Commercial humic acid; IAA, Indole-3-acetic acid (IAA).

The results of the analysis of variance of phosphorus and copper showed that there was no statistically significant difference between various levels of enriched HA treatments (Fig. 6a,b). However, application of CHA, IAA and HA-AS treatments showed the lowest amount of phosphorus and copper compared to the other treatments.

## Discussion

### Morphological traits

In the present study, foliar application of HA extracts and phytohormone IAA improve the growth characteristics of the canola plant. The increase in the plant growth features under foliar spraying of HA extracts or phytohormone IAA could be ascribed to the increase in the supply and root uptake of mineral source of nutrients which consequently created more favorable conditions for a better growth of canola plant^34,35^. The number of canola leaves in current experiments was affected by IAA treatment and had a significant increase compared to other treatments. In this context, Mir et al.^36^ showed the positive effects of foliar spraying of IAA hormone in improving the price and quality of *Brassica juncea* leaves at a concentration of 10^–8^ M, which was in line with the results of our research. Indole acetic acid as a phytohormone in suitable concentration had positive significant effects on plant growth^37,38^. It has been reported that IAA improves vegetative growth in plants by increasing cell division, photosynthesis and chlorophyll accumulation, and its positive effects on plants such as rice and maize have been determined by increasing leaf area and number, leaf diameter and plant height^39,40^. Canola plant height and stem diameter in our experiment were affected by HA extracted from NPS-enriched VCs (HA-NSP) as well as HA extracted from *Pseudomonas* and *Aztobacter* enriched VC (HA-AS). The results of our experiment showed that the use of the above-mentioned HA extracts increases the growth indices in the plant as similarly reported by other researchers^41–43^. Also, in the experiment conducted by Lally et al.^44^ on canola plant, similar to our research results, it was found that the use of *Pseudomonas* and *Aztobacter* can increase the height by 5.99%. Regarding the stimulating effects of HA extracts, many other studies have reported similar results on other plants. For example, foliar application of HA at the highest concentration (6%) increased the quality, growth and yield of statice cut flowers^45^. HA has been used on cucumber and has improved the nutritional balance and vitality of cucumber^46^. It has also been reported that with the use of humic substances, cell elongation increases, although this effect has been reported in low concentrations^47,48^. In addition, with the use of humic substances, due to their quasi-hormonal properties, the absorption of nutrients, especially nitrogen, and cucumber plant growth has been significantly stimulated^49^. In addition, in the present study, we have found that HA extracted from enriched VC had higher performance than CHA and IAA on some morphological traits such as height, stem diameter and leaf area. Similarly, some studies have focused on the comparison of HA extracted from VC on several crops such as strawberries^14^ and cucumber^50^. Arancon et al.^14^ investigated the effects of HA extracted from VC on strawberries plants and comparing them with the behavior of CHA and combined HA with plant growth hormones and IAA. They have shown that among these treatments, HA extracted from VC had the highest effect on the tested plants’ yield. Furthermore, it has been observed that with the increasing consumption of HA extracted from VC, plant root growth and the number of fruits have also increased. Atiyeh et al.^50^ reported that plant growth has a specific correlation with the increasing concentration of HA extracted from VC; nevertheless, this correlation depends on plant species, sources of VC, and the nature of plant typical content. They also believe that there was a direct relationship between the growth of tomatoes and cucumbers (*Cucumis sativus*) with 50–500 mg L^−1^ HA treatment, but as the HA concentration increases, for example, the concentration of 500–1000 mg L^−1^, plant growth decreases significantly.

### Leaf area, SPAD index, stomatal conductance and photosynthesis

Regarding the effects of treatments enriched with *Pseudomonas* and *Azotobacter* bacteria, it was found that they had the best effect on traits such as leaf area and SPAD index in the present study. Additionally, the use of CHA and IAA treatments as foliar application were not efficient enough in improving leaf area and SPAD value. In line with the results of this research regarding the Spad index, Ahmad et al.^51^ had similar results to ours, in such a way that the treatment of these bacteria caused a 37% increase in Spad index of mung bean (*Vigna radiata*) plant. In a study performed on grapes, Astaraei^52^ showed that humic substances contributed to the increase of leaf area. In addition, the use of endophytic bacterial strains to improve the growth and yield of plants, especially in tomatoes, has also been reported^53^. All this confirms the results of our study in the development of canola leaves. The results obtained on the effects of bacteria on the plant showed that there is a synergistic relationship between *Azotobacter* and *Pseudomonas* that leads to better use of soluble phosphorus sources and it also increases dry matter accumulation, grain yield and phosphorus uptake by wheat plants. The Synergistic relationship between *Azotobacter* and *Pseudomonas* leads to increased plant phosphorus in the soil, biological stabilization of nitrogen, increased availability of some trace elements such as iron and zinc, and production of plant growth promoters^54,55^. In plants such as African maize, the assimilation and uptake of nutrients by plant roots are largely controlled by the two main factors which are the leaf area and photosynthesis in each unit of leaf area, and with the increase of these two factors, plant growth and yield also increase^56^. Numerous studies have showed that complex compounds are formed between humic substances and mineral ions during the humification process, which increase the stimulation of enzymes and their effect on increasing the intensity of respiration, photosynthesis and metabolism of nucleic acid, and the quasi-hormonal activity of humic substances^57,58^. In the case of the positive effects of HA treatments extracted from VC on the canola growth parameters such as stomatal conductance and photosynthesis, it could be concluded that the humic substances cause positive effects on the photosynthesis efficiency of canola plant^59^. Also, in investigating the effects of HA in tobacco (*Nicotiana tabacum*), similar effects were reported and characterized by a significant increase in stomatal conductance and photosynthesis^60^. In addition, the photosynthetic activity of the lettuce plant (*Lactuca sativa* L.) increased with HA application due to the enhancement of chlorophyll content and mesophyll conductance^61^. Generally, HA can stimulate N metabolism and photosynthesis activity of plant which eventually improve biomass yield. In principle, it should be noted that photosynthesis in all plants is influenced by internal factors and environmental conditions. Internal factors of each cultivar determine the potential capacity of photosynthesis. Two of the internal factors affecting the rate of photosynthesis are sufficient amount of leaf chlorophyll and the leaves being healthy, which increase the rate of photosynthesis if the leaves are fresh^62^. Various environmental factors such as water availability, suitable moisture conditions, lack of water stress and high leaf water potential directly or indirectly affect the rate of photosynthesis^63^. There is a direct relationship between stomatal conductance and photosynthesis, and as stomatal conductance increase leads to improved photosynthesis. The parallel changes in photosynthesis rate and stomatal conductance indicate that the maintenance of photosynthesis can be attributed to the maintenance of stomatal conductance^64^. It has been reported that the increase in chlorophyll content is a reason for the increase in photosynthesis, and according to the results of SPAD, it can be considered as a factor for increasing photosynthesis^65^. Moreover, HA will increase the photosynthetic activity of the almond plant by increasing the activity of rubisco enzyme^66^. Based on the information mentioned, the main reason for the increase in HA in photosynthesis can be considered the increase in stomatal conductance in the leaves. According to its quasi-hormonal properties and growth stimulation mechanism^58^, it can be said that HA increases stomatal conductance and consequently increases photosynthesis. The reason for the increase in the HA extracted from VC compared to the CHA can be due to the origin of HA, more nutrients, aliphatic and aromatic compounds and more functional groups. In addition, one of the most important reasons for the superiority of these treatments (AS and NSP) compared to the other extracted HA treatments is the type of enrichment. *Pseudomonas and Azotobacter* bacteria have significantly increased the amount of phosphorus and nitrogen available to the plant, which affects plant growth and an increase in plant growth indices will be seen.

### Dry and wet weights of aerial organ and root, seed performance and inflorescence lengths

Foliar application of CHA and IAA on the treatments could not have a significant effect on the fresh and dry weights of canola aerial organs and roots. However, HA extracted from VC enriched with nitrogen, sulfur, and phosphorus (HA-NSP) was the best treatment. A similar result in enrichment with these three important elements has also been observed on canola^3^. The positive effect of enriched VC with nutrients such as nitrogen and phosphorus were similar to Kumar and Singh^67^ and Mahanta et al.^68^, who reported that enrichment increased the availability of nitrogen and subsequently rice growth. Sharma et al.^69^ reported that with increasing nitrogen availability around the root and/or applying as a foliar spray on the plant surface in the early stages of growth, the common bean shoots significantly increased. Also, Turk et al.^70^ revealed that application of phosphorus fertilizer caused increases root length, fresh and dry weight of the lentil (*Lens culinaris Medik*) plant. Daur and Bakhashwain^71^ found that with the application of humic substances under controlled conditions, the dry weight of corn (*Zea mays*) yield and oat (*Avena sativa*) seedlings increased significantly. In another experiment, the effect of foliar application of HA and nitrogen on durum wheat was investigated, and the results showed that HA caused a significant increase in the dry weight of wheat stalk and root^72^. The stimulating effects of HA extracted from VC on dry weight of shoot and root was similar to the work of Celik et al.^73^, who reported that foliar application of HA significantly increased the dry weight and mineral elements uptake by maize roots (*Zea mays*). In an experiment done on wheat, it was found that the foliar application of 54 mg per liter of HA resulted in a 50% increase in root length and a 22% increase in dry matter, as well as a significant increase in nitrogen uptake in the presence of HA^42^. In a greenhouse experiment, researchers studied the effect of HA on fresh and dry weight and oat yield and found that by applying 20 kg of HA per pot, the fresh and dry weight of the *Achillea millefolium* L. increased significantly^74^.

In general, foliar application of HA significantly increases the concentration of antioxidants and increases photosynthesis, respiration, nucleic acid synthesis, and ion uptake by roots^75^. In this regard, an interesting relationship was found between photosynthesis rate and grain weight in this experiment, which could be related to the increase in grain yield connected to the enhanced photosynthesis and stomatal conductance in the mentioned treatments. In this experiment, the seed performance of canola was significantly affected by HA-AS treatment. Phosphorus is one of the main nutrients required by the plant and affected the production of its yield. It has also involved in all biochemical processes and the transfer of photosynthetic products^76,77^. *Pseudomonas fluorescens* is one of the most important phosphate-solubilizing microorganisms. These bacteria directly contribute to better plant growth via stimulate plant growth through physiological mechanisms such as the production of plant hormones and the dissolution of phosphate^17^. Also, *Azotobacter* has a positive effect on plant yield by fixing nitrogen directly^78^. Therefore, the availability of phosphorus and nitrogen through the use of this treatment (HA-AS) on canola seed yield was proven in this experiment. It has also been reported that the use of a mixture of *Azotobacter* with *Pseudomonas* has increased the number of seeds per pod and increased the weight of sorghum (*Sorghum bicolor* L.) seeds^79^. In continuation, according to the reports of some researchers, it has been found that the presence of sufficient phosphorus and nitrogen in plants increases the number of seeds and seed weight per plant^77,80^. On the other hand, it has been shown that the use of HA has a positive effect on the reproductive growth of canola, followed by an increase in seed weight and the number of pods^81^. Due to the hormonal-like effects of HA and also by providing conditions to increase the uptake of nutrients from the roots, HA increases plant growth, height of plant and inflorescence length^58^, which was consistent with the results of our research. Other researchers have confirmed the increase in inflorescence length with the use of HA^82,83^.

### Oil percentage in seeds and concentrations of phosphorus and copper in the aerial part of canola

When looking at the oil percentage difference in the present study, no significant effect was observed between the implemented treatments (Fig. 5). However, in contrast to the results of our research, Nasiri et al.^84^ by investigating the effect of HA foliar application on canola plants, found that the use of HA at a concentration of 3/1000 [v/v] caused an increase of up to 5.4% in the amount of oil compared to the control treatment. The amount of total oil in canola was reported to be 40–45%^85^, which was not similar to the results of the present study (36%). Greenhouse cultivation conditions and lack of fertilization during the growing season can be the reason for the lack of oil existing in canola compared to the previous reports. Even if the amount of seed oil is a genetically inherited trait, the environmental conditions are also playing a role. Among the environmental factors that affect the amount of oil, temperature is the most important factor that decreases the percentage of oil^86^. Therefore, to some extent, the lack of effect of HA on the percentage of canola (*Brassica napus*) oil in this experiment can be explained. According to the results of the current study, HA increased the absorption of copper and phosphorus, which is consistent with the results of other studies^87,88^. The use of HA also increased the mineral content of the leaves of various plants. It can be said that this acid has direct and indirect effects on wheat plants, and its use accelerates the plant growth and uptake of nutrients^89^. Moreover, by the use of HA treatment, the absorption of micro and macro elements in the wheat plant is increased^90^. It seems that the positive effects of foliar application of HA, such as the effects of its soil application, are due to the presence of quasi-hormonal compounds from the group of auxins, cytokinins and gibberellins in the grain plant. Considering the results of different studies, it seems that according to the origin of the HA used, its concentration, substrate type and pH, as well as how it is used are effective factors in the results obtained^91^. In addition, in this study, foliar application treatments were effective in absorbing copper and phosphorus elements, based on which it can be said that the use of foliar spraying is a rapid method of storing microelements since many micronutrients are stabilized by most soils. Regarding macro elements, it is important to note that HA can increase root access to these elements by chelating calcium and magnesium elements in the soil^92^. The use of HA increases the absorption of nutrients and the ability to obtain soil nutrients, especially in alkaline soils with low organic matter^19^. This combination also increases the mobility and transport of macro and micro elements and the ability to use micronutrients^43^. It should be mentioned that HA indirectly increases phosphorus uptake through the formation of complex compounds with iron^93^. For instance, a study conducted on the effect of several types of HA on the activity of root phosphatase enzyme showed that HA increases the uptake of phosphorus in wheat plant by combining and forming a complex with phosphatase enzyme^93^. The use of HA increases the ability of wheat plant to retain more nitrogen content along with other micro and macro elements^94,95^. Humic substances extracted from VC increase the absorption of elements such as magnesium, copper, phosphorus and nitrogen in *Festuca arundinacea*^96^. All morphological traits, yield, and the content of NPK macronutrients in kidney bean plant (*Phaseolus vulgaris*) tissue and chlorophyll content were significantly increased by the application of HA along with amino acid in beans^97^. Furthermore, foliar application of HA has increased the uptake of copper, zinc, phosphorus, potassium, magnesium and sodium in corn^90^.

## Conclusion

Growth promotion effects activated by sprays of humic acid (HA) extracted from enriched vermicompost, commercial HA (CHA) and indole acetic acid (IAA) on canola plant is indicating a growth stimulation effect under controlled conditions.

The point in hope in this study was the high performance of bacteria-enriched treatment, which was often similar to the treatment enriched with chemical fertilizers. The variety of treatments that existed in this experiment were able to show significant differences among traits. For example, among the HA treatments extracted from enriched VC, the treatment enriched with 3 important elements: nitrogen, sulfur and phosphorus (HA-NSP) and/or *Azotobacter chroococcum* + *Pseudomonas fluorescens* (HA-AS) had the best effect on growth characteristics (height, stem diameter, leaf area and SPAD index) and yield of canola. Also, HA-N were the less influencing treatments among VC enrichment treatments. On the other hand, HA extracted from VC had significantly better results than commercially used HA in most growth parameters for similar treatment concentrations. Another aim of this research was to compare HA extracted from VC with IAA in terms of hormonal properties. In this study, we found out the hormonal properties of HA and IAA are not the same and it is not possible to make an accurate assessment of the stimulating effects of these substances on plant growth. In this regard, it is suggested to other researchers for further studies to investigate the hormonal properties of HA with other hormones and also in different concentrations. On the other hands, there are some limiting factors in commercialization and large-scale application of HA extracted from VC including high enrichment costs and incubation period and low amounts of HA in VC. Therefore, the production of VC with materials containing high HA and also doing enrichment operation from the beginning of the VC production process can be suggested in the future study.

## Data Availability

The datasets used or analyzed during the current study are available from the corresponding author on reasonable request.
